# Reversible, Chemically Gated FRET via Ligand‐Activated Acceptors

**DOI:** 10.1002/advs.77693

**Published:** 2026-09-27

**Authors:** Nivedita Singh, Smruti Ranjan Nayak, Sanjay Kumar Mohanty, Aishwarya Hanumantharaju, Amala Shaju, Mini Jose, Aravind Penmatsa, Deepak Nair

**Affiliations:** ^1^ Centre For Neuroscience Indian Institute of Science Bangalore India; ^2^ Molecular Biophysics Unit Indian Institute of Science Bangalore India; ^3^ Cell and Tissue Imaging Center‐Light Microscopy St Jude Children's Research Hospital Memphis USA

## Abstract

Genetically encoded fluorogen‐activating tags enable conditional fluorescence, yet the structural and excited‐state mechanisms underlying ligand activation remain unclear. Here, we combine X‐ray crystallography, computational modelling, and fluorescence lifetime imaging microscopy (FLIM) to define the structural and photophysical basis of fluorogen activation in the Fluorescence‐Activating and Absorption‐Shifting Tag (FAST). Structures of apo and ligand‐bound FAST reveal the interface for fluorogen binding. In the apo state, N‐terminal residues occlude the pocket, whereas ligand binding displaces these elements to generate a solvent‐accessible cavity stabilized by ordered water molecules. Distinct hydroxybenzylidene rhodanine derivatives differentially tune interfacial geometry and excited‐state ensembles, producing single or multiexponential fluorescence lifetimes in living cells that primarily reflect modulation of excited‐state relaxation. Leveraging this ligand dependence, we utilize FAST as a chemically gated Förster resonance energy transfer (FRET) acceptor that induces reversible, concentration‐dependent donor lifetime shortening that does not require additional external controls. Using ligand‐controlled FLIM‐FRET, we resolve supramodular organization within Membrane‐Associated Guanylate Kinase (MAGUK) scaffolds in live cells, establishing FAST as a reversible photophysical module for mapping molecular architecture.

## Graphical abstract

Fluorogen binding turns the compact FAST tag into a tunable FRET acceptor, with ligand chemistry programming spectral overlap and excited‐state lifetimes. Fluorescence‐lifetime imaging reads out energy transfer through the shortened donor lifetime, while simply adding or washing out the dye switches FRET on and off, reversibly mapping the supramodular architecture or spatial heterogeneity of protein interactions in living cells.

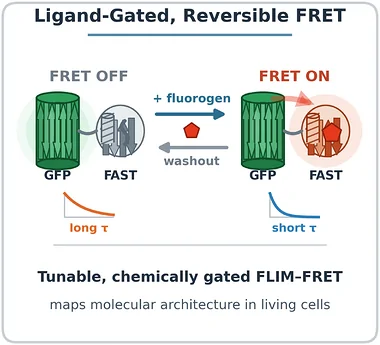

## 1 Introduction

Förster resonance energy transfer (FRET) is widely used to measure nanoscale proximity and conformational changes. However, conventional FRET systems typically rely on acceptors that are either constitutively fluorescent or undergo radiative decay upon excitation, leaving their photophysical properties largely predetermined and limiting dynamic, internally controlled measurements [1, 2, 3]. A stochastically activatable or ligand‐dependent acceptor would enable reversible, concentration‐controlled modulation of energy transfer within the same cellular context, allowing the same construct to serve as both sample and control [1, 4]. More broadly, fluorogen activating systems have already shown that energy transfer and fluorogenic output can be made conditional on fluorogen binding, donor sensitization, or proximity‐dependent ligand capture [4, 5, 6, 7]. Here we developed a strategy that requires ligand binding to produce a well‐defined excited‐state configuration with predictable spectral overlap with donor and lifetime changes reflecting redistribution between interacting and non‐interacting populations rather than intrinsic perturbation of donor photophysics [1, 8, 9]. These requirements are particularly stringent in large, multidomain scaffolding assemblies, where structural heterogeneity and supramodular organization complicate interpretation of FRET measurements. Membrane‐Associated Guanylate Kinase (MAGUK) scaffolds provide a representative example, organizing signalling complexes through their modular PDZ, SH3, and GUK domains [10, 11, 12, 13]. Their supramodular architecture arises from intramolecular domain coupling and higher‐order assembly [14, 15, 16, 17], yet resolving spatial relationships within these complexes in living cells remains challenging [18, 19, 20]. A reversible, chemically gated FRET module would therefore provide a powerful means to interrogate these assemblies without permanent spectral perturbation or stoichiometric constraints [1]. Here, X‐ray crystallography, computational modelling, and quantitative fluorescence lifetime imaging microscopy FLIM are combined to define the structural and excited‐state basis of fluorogen activation in FAST. In FAST, ligand binding remodels the interfacial architecture, while chemical substitution reshapes the excited‐state relaxation pathways. Together, these properties provide a basis for harnessing ligand engagement to design a reversible FRET acceptor. Ligand‐controlled FLIM‐FRET is further applied to probe supramodular organization within MAGUK scaffolds in living cells. By integrating structural chemistry with live‐cell photophysics, this work establishes a framework in which ligand identity programs excited‐state behavior and enables chemically gated control of energy transfer for mapping molecular architecture in complex cellular assemblies.

Genetically encoded fluorescent proteins have become indispensable tools for visualizing molecular organization and dynamics in living systems. However, canonical fluorescent proteins rely on a covalently matured chromophore embedded within a rigid β‐barrel scaffold, limiting chemical tunability and reversibility of optical output. Fluorogen‐activating proteins provide a complementary strategy in which fluorescence arises from reversible, noncovalent association with synthetic ligands. Because fluorogen is weakly emissive in solution and brightens upon binding, fluorescence becomes conditional on ligand engagement, enabling reduced background, temporal control, and chemical programmability [21, 22]. The Fluorescence‐Activating and Absorption‐Shifting Tag (FAST) is a compact (∼14 kDa) fluorogen‐activating protein that binds hydroxybenzylidene rhodanine (HBR) derivatives spanning a wide spectral range [22, 23, 24, 25]. Ligand binding is rapid and reversible, permitting dynamic labelling and fluorogen exchange in living cells [22, 23, 26]. Despite its versatility, the molecular logic by which FAST converts chemically diverse, weakly fluorescent ligands into bright emitters remains incompletely understood, although recent structural work has begun to define ligand‐induced ordering, binding pocket architecture, and fluorogen‐specific stabilization [27, 28, 29]. In particular, how ligand chemistry, interfacial binding geometry, and local solvation collectively shaped excited‐state energy landscapes have not been defined at structural resolution [27, 29, 30].

Fluorescence activation in fluorogen‐binding systems typically arises from restriction of intramolecular rotation, stabilization of planar conjugation, and modulation of excited state behavior that suppress non‐radiative decay [21, 30, 31]. FAST is distinctive in that ligand binding occurs such that the quaternary organization and conformational plasticity may directly influence excited‐state stabilization [27, 29]. Ligand binding restricts intramolecular rotation around the methine bridge, suppressing non‐radiative relaxation pathways, while the protein environment stabilizes emissive excited states through a network of polar, hydrogen‐bonding, and solvent‐mediated interactions. Together, these effects enhance fluorescence and remodel the excited‐state energy landscape, such that fluorescence lifetime reflects the integrated photophysical properties of the protein–ligand complex rather than the degree of rotational restriction alone. An additional open question concerns the relationship between ligand chemistry and excited‐state ensemble behavior in cells. HBR derivatives differ in substitution pattern, conjugation, and electronic distribution, features known to modulate fluorogen brightness, spectral position, and non‐radiative relaxation [24, 30, 32]. Such variations could bias the protein–ligand complex toward either homogeneous emissive states or heterogeneous excited‐state populations with distinct decay pathways, a possibility that aligns with recent fluorescence lifetime work on FAST variants and fluorogen combinations [8, 9]. To examine how fluorogen structure shapes excited‐state behavior, we selected three related HBR derivatives that differ only in aromatic substitution: HMBR, HBR‐3,5‐DM, and HBR‐3,5‐DOM. This series provides a systematic progression in electron‐donating strength and steric environment, leading to red‐shifted spectral properties and potentially altered constraints on fluorogen torsional motion within the FAST‐binding pocket. Determining how chemical substitution reshapes lifetime distributions in living cells is critical for rational photophysical tuning and for exploiting FAST as a quantitative reporter. The reversible nature of fluorogen binding further suggests a broader possibility of harnessing ligand engagement to regulate energy transfer rather than merely activating fluorescence [1, 5].

Beyond its specific application to FAST, the framework developed here addresses a general need in quantitative bioimaging. Most live‐cell imaging strategies rely on fluorophores whose photophysical properties are fixed once expressed, so that quantitative readouts such as fluorescence lifetime or FRET efficiency cannot be switched on or off, titrated, or internally calibrated within the same sample. By showing that the chemistry of a small, exchangeable ligand can be used to program excited‐state behavior and to gate energy transfer reversibly, we provide a generalizable design logic for environment‐sensitive, chemically controllable, and self‐calibrating optical reporters. Such reporters are broadly relevant to mapping protein localization, conformation, and interaction networks across cell biology, and are not restricted to the study of fluorogen‐activating proteins themselves.

## 2 Results

### 2.1 Structure of FAST Domain‐Swapped Dimer

**FIGURE 1:**
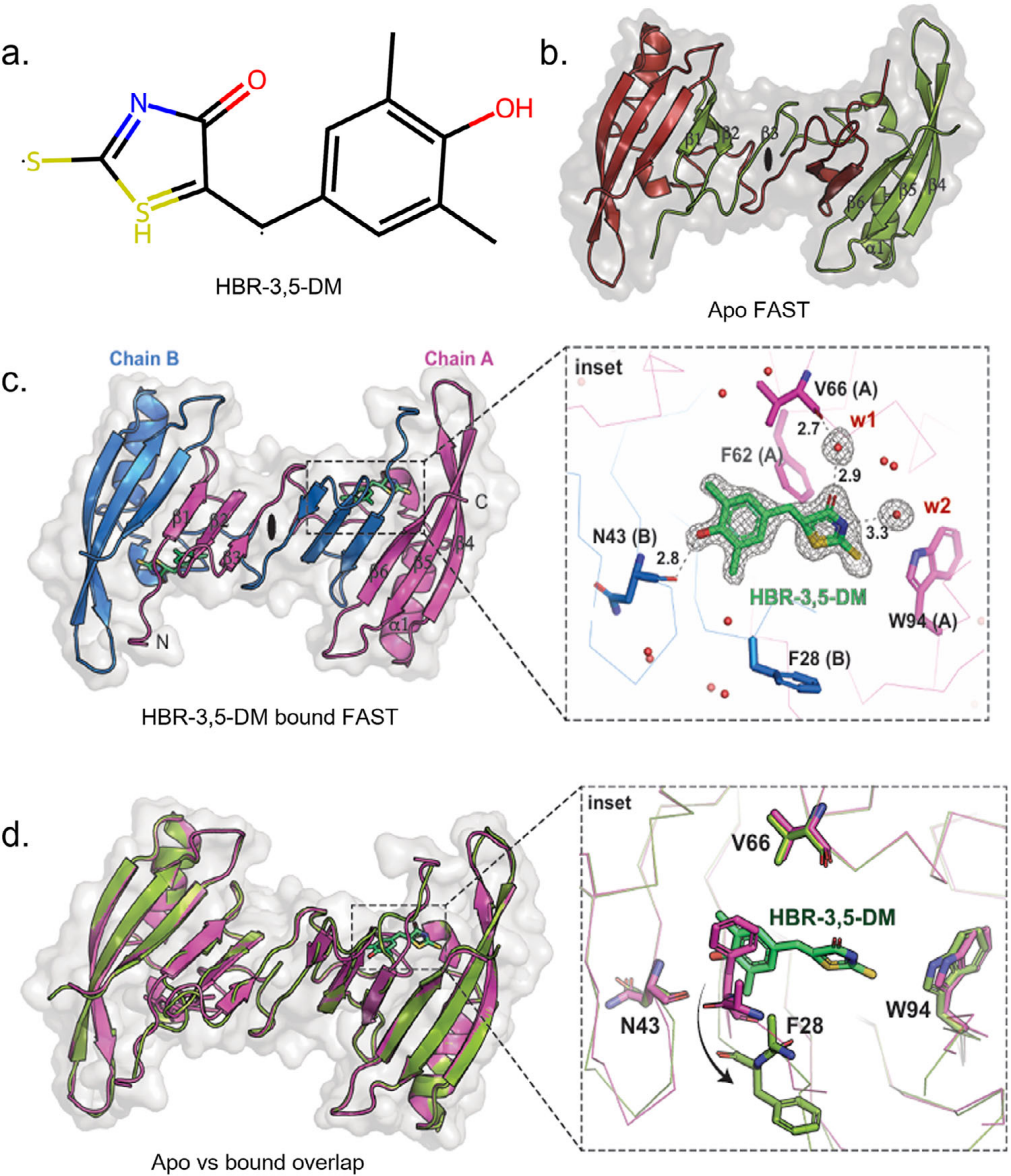
Structural Basis of Ligand Activation in FAST. (a) Chemical structures of the fluorogen HBR‐3,5‐DM. (b) Dimeric structure of apo form of FAST displays a domain‐swapped dimer, as observed in the crystal structure. The symmetry‐related protomer (brick) is related by a twofold symmetry (black oval). The individual secondary structure motifs are represented. (c) HBR‐3,5‐DM (green) bound structure displays the binding site at the interface of the two protomers. Inset shows the Fo–Fc density of the fluorogen contoured at 3.0σ along with two waters (red) that solvate the HBR‐3,5‐DM in the binding pocket. Residues in the vicinity from the two chains are shown and coloured as per the contributing chain. Numbers at dashed lines indicate distances in angstroms (Å). (d) Structural overlap of the apo and fluorogen‐bound structures (Cα–Cα rmsd = 0.32 Å) displays the movement of the N‐terminal β‐bulge residues that fill the binding pocket occupied by HBR‐3,5‐DM in the apo state.

The X‐ray structures of the fluorogen free FAST (apo) and FAST bound to HBR‐3,5‐DM were determined to resolutions of 2.9 and 1.6 Å, respectively (Figure 1a–d, Tables S1 and S2). The structure of single protomers of FAST was determined in a tetragonal lattice similar to the previously reported structure of the FAST protein without bound ligands [29]. Despite a single protomer structure being refined, the structure of FAST showed a predominant β‐sheet containing molecule with two distinct N‐ and C‐terminal domains connected by a long linker from residues 51 to 76. The two distant domains formed a domain‐swapped dimer with a symmetry related equivalent in the crystal lattice, leading to formation of an obligate dimer in solution for the FAST protein. The domain swapped dimer shared an interfacial area of 1936 Å^2^ which constituted about 25% of the solvent accessible surface area of the individual protomers. The N‐terminal domain was a three antiparallel β‐strand containing motif that stacked with the C‐terminal domain β‐strands of the symmetry‐related protomer to establish a 6 strands antiparallel β‐sheet, with the N‐ and C‐termini being in close proximity in the dimeric protein. In the apo form of the FAST protein, a deviation in the strands between residues 25 and 29 allowed the formation of a β‐bulge, particularly at residues L26, A27 and F28 that form a hydrophobic region that occludes solvent access to the core of the dimer. Surprisingly, in our structure we observed partial density where this stretch was exposed, and an alternate conformation for this region of the N‐terminus, which was dynamic in the FAST protein (Figure 1b). The HBR‐3,5‐DM bound FAST displayed a similar domain swapped dimer organization, where the binding pocket for HBR‐3,5‐DM was sandwiched by four β‐sheets and the central helix from one protomer and the N‐terminal strands of the domain‐swapped protomer. Given the high resolution of this structure obtained in this study (1.6 Å), we could clearly model and observe densities for this fluorogen and its surrounding residues. Interestingly, the β‐bulge observed in the apo structure was displaced by the binding of the HBR‐3,5‐DM in the interface of the two protomers, suggesting an induced fit during binding. The fluorogen's position exactly overlapped with the F28 residue position in the apo state, that was displaced by the binding of HBR‐3,5‐DM with nearly 9.0 Å movement at the Cγ‐position of F28. The fluorogen interacted with residues W94, V66, F6, and F28. The hydroxy group of the 3,5 dimethyl phenol group of the fluorogen interacted with the main chain carbonyl of the N43 from the domain‐swapped protomer, holding the fluorogen in place. The N‐atom of the thioxothiazolidin moiety interacted with a water molecule at a distance of 3.3 Å. Similarly, the O‐atom in the same moiety interacted with another water molecule at a distance of 2.9 Å making the environment around the fluorogen amphiphilic and prone to spectral changes through hydration and changes in the hydrophobic environment (Figure 1c,d).

**FIGURE 2:**
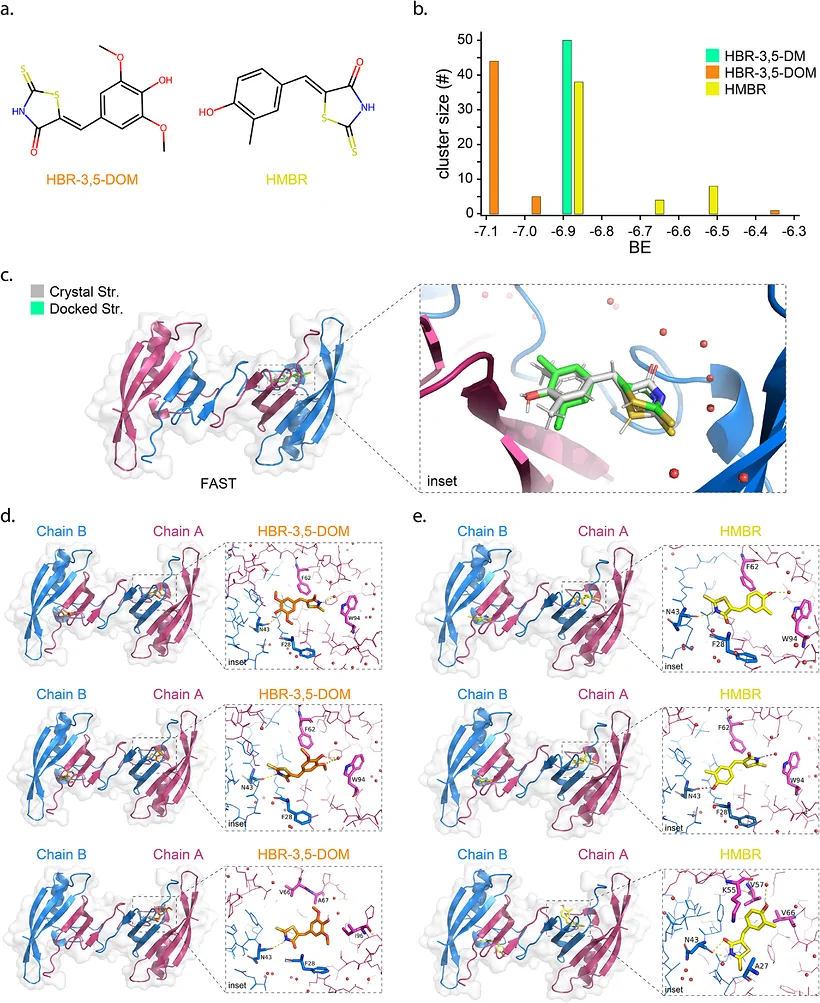
Divergent Ligand Engagement Drives Distinct FAST Protomer Conformations. (a) Chemical structures of the fluorogens HBR‐3,5‐DOM and HMBR. (b) Barplot illustrating binding mode cluster size (out of 50) on the y‐axis versus the binding energy (BE) kcal/mol of the corresponding cluster representative on the x‐axis. (c) Inset showing a comparison of the HBR‐3,5‐DM binding mode between the crystal structure (grey) and the docking model (green). (d) Representative 3D complexes with HBR‐3,5‐DOM (orange) docked at the interface of the two protomers. The inset highlights the corresponding binding mode, including the interacting residues and water molecule (red). (e) Representative 3D complexes with HMBR (yellow) docked at the interface of the two protomers. The inset highlights the corresponding binding mode, including the interacting residues and water molecule (red).

As the complexes between HBR‐3,5‐DOM or HMBR‐bound FAST did not yield crystals of good quality, we used the crystal structure of HBR‐3,5‐DM‐bound FAST for in silico analysis to evaluate and compare the interaction patterns of HBR‐3,5‐DOM and HMBR (Figure 2a). We cross‐validated the approach by performing molecular docking of HBR‐3,5‐DM against FAST to determine whether we could reproduce the same binding mode observed in the crystal structure. In silico docking of HBR‐3,5‐DM, HBR‐3,5‐DOM and HMBR against FAST generated 50 protein‐ligand complexes for each ligand. Clustering of the docked poses based on ligand orientation within the complex resulted in three distinct clusters each for HBR‐3,5‐DOM and HMBR, whereas HBR‐3,5‐DM produced a single cluster (Figure 2b). For subsequent analysis, the conformation with the highest binding affinity (i.e., lowest binding energy) within each cluster was selected as the representative structure. HBR‐3,5‐DM exhibited a root mean square deviation (RMSD) of ∼0.8 Å between its binding mode in the resulting docked complex and that in the crystal structure, demonstrating that the in silico approach was capable of reproducing the experimentally observed binding mode (Figure 2c). HBR‐3,5‐DOM exhibited a highly dominant binding conformation. The largest cluster contained 44 out of 50 (88%) poses, with a representative binding energy of −7.08 kcal/mol (Figure 2c, Figure S1a). The remaining two clusters contained 5 and 1 (12%) poses, with representative binding energies of −6.97 and −6.35 kcal/mol, respectively (Figure 2b,d, Figure S1a). As the representative binding energies of the three clusters span only ∼0.7 kcal/mol, which is comparable to the thermal energy available at room temperature (RT ≈ 0.6 kcal/mol) and well within the intrinsic uncertainty of the scoring function, these energy differences cannot be interpreted as evidence of thermodynamic exclusivity between binding modes. Instead, the strong preference for a single binding geometry is inferred from the convergence of the independent docking trials: 88% of the 50 stochastic runs converged to one cluster, which reflects the relative width and accessibility of that free‐energy basin rather than a large energy gap. As shown below, this computational inference is independently corroborated by the monoexponential fluorescence decay measured for FAST::HBR‐3,5‐DOM in live cells. In contrast, HMBR displayed a more distributed clustering pattern, with 38 (76%), 8 (16%), and 4 (8%) poses in the three respective clusters (Figure 2b,e, Figure S1b). The representative binding energies for these clusters were −6.86, −6.65, and −6.51 kcal/mol (Figure 2b,d, Figure S1b). The relatively balanced distribution of conformations suggests more than one plausible interaction configuration for HMBR within the FAST binding pocket. Furthermore, among the interacting residues, N43 exhibited the strongest interaction, forming a hydrogen bond compared to the predominantly hydrophobic interactions observed for other residues. This interaction was consistently maintained across all fluorogens. The fluorogens also showed stable interactions with F28, which occupies the binding space in the apo structure, thereby identifying both N43 and F28 as key residues involved in binding. In addition, all fluorogens displayed interactions with water molecules, which could likely contribute to spectral changes through hydration effects and alterations in the local environment.

### 2.2 Fluorogen‐Dependent Photophysics of FAST in Live Cells

**FIGURE 3:**
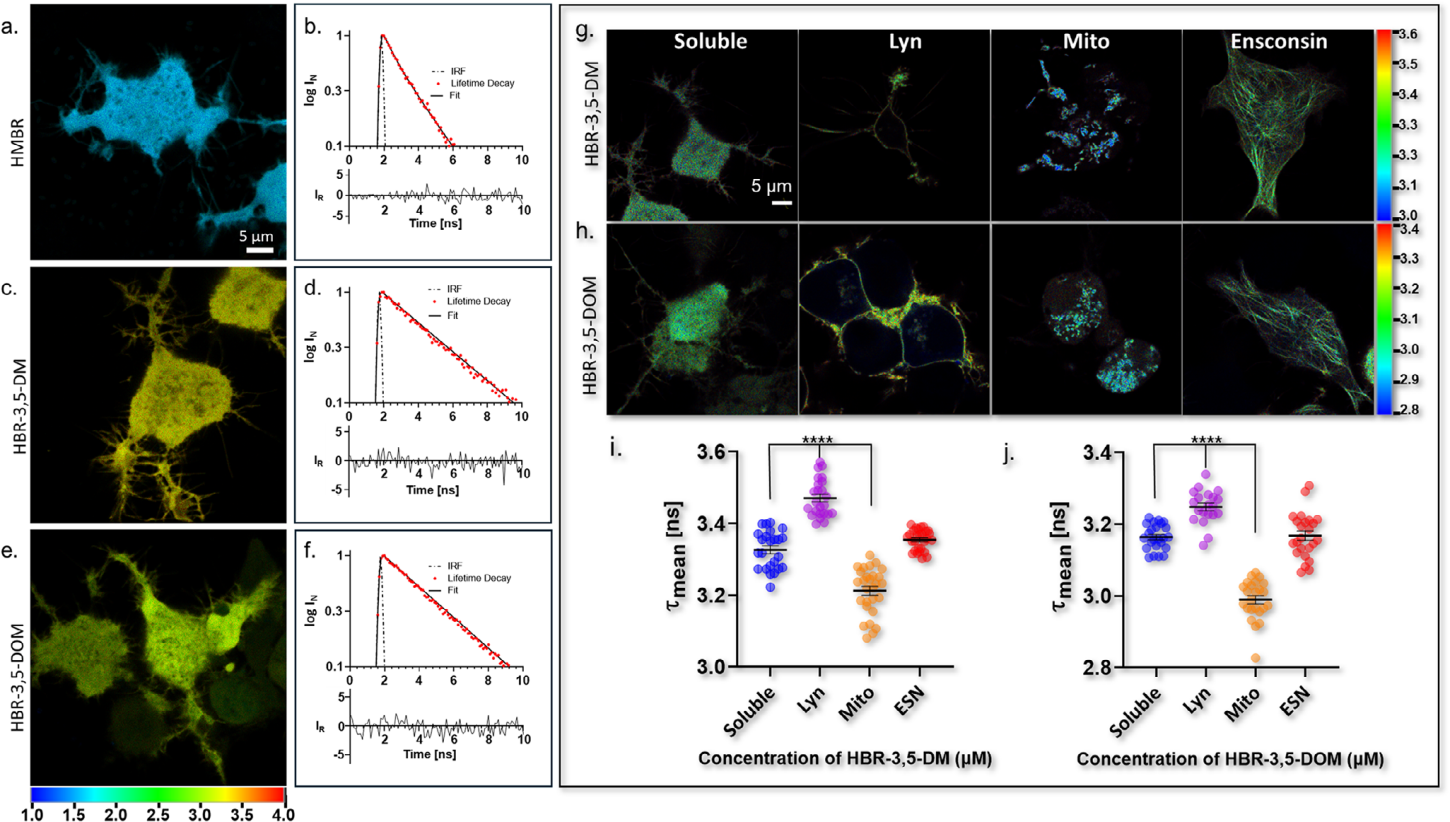
Ligand‐Dependent Fluorescence Lifetime Dynamics of FAST in Neuro‐2a Cells. (a, c, e) FLIM images of Neuro‐2a cells expressing cytoplasmic FAST labelled with HMBR (a), HBR‐3,5‐DM (c), and HBR‐3,5‐DOM (e). FLIM images are represented in a pseudocolour scalebar range 1.0–4.0 ns. (b, d, f) Fluorescence intensity decay of the FAST‐fluorogen complexes. HMBR and HBR‐3,5‐DM were excited at 488 nm, while HBR‐3,5‐DOM was excited at 518 nm. HMBR exhibited a biexponential decay profile, indicating a heterogeneous population with a mean lifetime of 1.48 ± 0.09 ns (b), while HBR‐3,5‐DM (d) and HBR‐3,5‐DOM (f) showed monoexponential decay, representing a homogeneous population with a mean lifetime of 3.33 ± 0.04 ns for FAST::HBR‐3,5‐DM and 3.13 ± 0.03 ns for FAST‐HBR‐3,5‐DOM. Data represent mean ± SD from at least three independent experiments (≥12 cells per condition). Scale bar: 5 µm. (g) FLIM images of Neuro‐2a cells expressing soluble FAST and subcellularly localized FAST constructs, Lyn‐FAST (membrane‐targeted), Mito‐FAST (mitochondria‐targeted), and Ensconsin‐FAST (microtubule‐associated) in presence of 10 µM HBR‐3,5‐DM. FLIM images are displayed using a pseudocolour scale ranging from 3.0–3.6 ns. (h) FLIM images of the same constructs in presence of 10 µM HBR‐3,5‐DOM, represented with a pseudocolour scale ranging from 2.8‐3.4 ns. (i) Comparison of amplitude‐weighted mean fluorescence lifetimes (ns) of soluble FAST, Lyn‐FAST, Mito‐FAST, and Ensconsin‐FAST in the presence of 10 µM HBR‐3,5‐DM (left) and (j) 10 µM HBR‐3,5‐DOM (right). Lyn‐FAST showed a significant increase in mean lifetime, while Mito‐FAST displayed a significant decrease compared to soluble FAST. Ensconsin‐FAST remained unchanged. Data represent mean ± s.e.m. from at least three independent experiments (≥20 cells per condition). Scale bar: 5 µm.

To evaluate the photophysical behavior of the FAST‐fluorogen system and its suitability for fluorescence lifetime imaging microscopy (FLIM), we performed volumetric imaging in live Neuro‐2a cells expressing cytosolic FAST. Imaging was conducted on a confocal microscope equipped with time‐correlated single‐photon counting (TCSPC), providing picosecond resolution of fluorescence decay kinetics. System calibration was first performed using two reference fluorophores, Rhodamine B (*τ* = 1.45 ± 0.06 ns) and Coumarin 6 (*τ* = 2.45 ± 0.02 ns; Figure S2a–f) [33, 34]. Both reference standards displayed monoexponential decays, and phasor representation localized each fluorophore on the universal semicircle, confirming the accuracy of the TCSPC module and the reliability of analytical workflow. Using the same acquisition conditions, cytosolic GFP expressed in Neuro‐2a cells was imaged as a cellular fluorescence reference. GFP exhibited a monoexponential decay (*τ* = 2.45 ± 0.02 ns; Figure S3a–d) with a tightly clustered phasor distribution, reflecting homogeneous chromophore environments and validating the experimental setup and analytical workflow. Live‐cell FLIM of FAST labelled with HMBR (10 µM) revealed markedly shorter lifetimes and non‐monoexponential decay profiles (Figure 3a,b, Figure S3e–h). Biexponential fitting resolved two dominant emissive species (*τ*_1_ = 0.65 ± 0.08 ns, *τ*_2_ = 1.84 ± 0.04 ns), contributing 36 ± 2% and 64 ± 2% to the total intensity, respectively. In addition, HMBR exhibited transient binding states to the FAST protomer, with one state accounting for 76% of the population and other closely spaced states together contributing 24% of the FAST::HMBR complex. The resulting broadened and displaced phasor distribution reflects a heterogeneous population, consistent with conformational flexibility of the bound chromophore observed through molecular docking studies (Figure 2a) and a dynamic equilibrium between long‐ and short‐lived emissive states. To systematically compare fluorogen effects, we measured lifetimes for FAST complexes with HBR‐3,5‐DM and HBR‐3,5‐DOM (10 µM each). Both complexes exhibited monoexponential decays (*τ* = 3.33 ± 0.04 ns and 3.13 ± 0.03 ns, respectively), indicative of reduced chromophore flexibility and efficient suppression of non‐radiative relaxation pathways (Figure 3c–f). These observations are consistent with crystallographic data, in which HBR‐3,5‐DM adopts a single planar geometry within the binding pocket maximizing *π*‐conjugation and electronic coupling to the protein scaffold. In silico observation indicated that HBR‐3,5‐DOM also displayed transient binding states to the protomer; however, two closely spaced states in binding energy together accounted for 98% of the FAST::HBR‐3,5‐DOM complex (Figure 2a). Fluorogen titration across 1.25–10 µM showed minimal variation in mean lifetimes (Figure S4, Table S3), confirming stable, reversible binding and negligible influence from unbound species. Since HMBR in complex with FAST in living cells consistently presented biexponential decays, we focused our attention on HBR‐3,5‐DOM and HBR‐3,5‐DM. The invariant lifetime and long, monoexponential decays highlight the high photostability and homogeneity of HBR‐3,5‐DM and HBR‐3,5‐DOM. Finally, we investigated how subcellular localization modulates FAST photophysics. Constructs targeting cytosol, plasma membrane (Lyn‐FAST), mitochondria (Mito‐FAST), and microtubules (Ensconsin‐FAST) were imaged with HBR‐3,5‐DM or HBR‐3,5‐DOM. Lifetime maps revealed small but significant patterns distinctly reflecting local environments: Mito‐FAST exhibited the shortest lifetimes, consistent with quenching in the high‐polarity mitochondrial matrix; Lyn‐FAST displayed slightly longer lifetimes, suggesting reduced solvent exposure at the membrane; and Ensconsin‐FAST matched cytosolic FAST, indicating minimal chromophore perturbation upon microtubule binding (Figure 3g–j, Table 1, Table S4). Monoexponential decays were observed across compartments, with each FAST–fluorogen complex predominantly exhibiting a predominant resolvable emissive species, consistent with the chemical stability and reproducibility of the transient FAST–ligand complex. As HBR fluorogens are molecular rotors, their fluorescence lifetime is sensitive to the local physicochemical environment, particularly factors that influence torsional relaxation, such as microviscosity, polarity, and electrostatics. Accordingly, the shorter lifetimes observed in mitochondria and the longer lifetimes at the plasma membrane likely reflect differences in these combined environmental properties rather than changes in polarity alone. Overall, these results establish FAST as a versatile platform for quantitative FLIM, with fluorogen‐dependent photophysics, environmental sensitivity, and reversible binding enabling precise mapping of protein localization, conformational states, and nanoscale cellular heterogeneity. The FAST‐HBR‐3,5‐DM complex, in particular, represents a benchmark probe for lifetime‐based biosensing and dynamic imaging in live cells.

**TABLE 1:** Fluorescence lifetime of different FAST constructs in live Neuro‐2a cells. It shows the median (IQR) and mean ± SD fluorescence lifetime values of soluble FAST, Lyn‐FAST, Mito‐FAST, and Ensconsin‐FAST constructs measured in live Neuro‐2a cells in the presence of 10 µm HBR‐3,5‐DM and 10 µm HBR‐3,5‐DOM individually.

	Soluble FAST	Lyn FAST	Mito FAST	Ensconsin FAST
HBR‐3,5‐DM (ns)	3.34 (3.28‐3.37)	3.46 (3.43‐3.52)	3.23 (3.17‐3.26)	3.36 (3.33‐3.38)
	3.33±0.05	3.47±0.05	3.21±0.06	3.36±0.03
HBR‐3,5‐DOM (ns)	3.16 (3.14‐3.20)	3.25 (3.23‐3.28)	3 (2.96‐3.03)	3.16 (3.12‐3.21)
	3.16±0.04	3.25±0.05	2.99±0.06	3.17±0.06
				Median (IQR)
				Mean ± SD

### 2.3 FAST‐Fluorogen as a Tunable, Reversible FRET Acceptor with Intrinsic Baseline Controls

**FIGURE 4:**
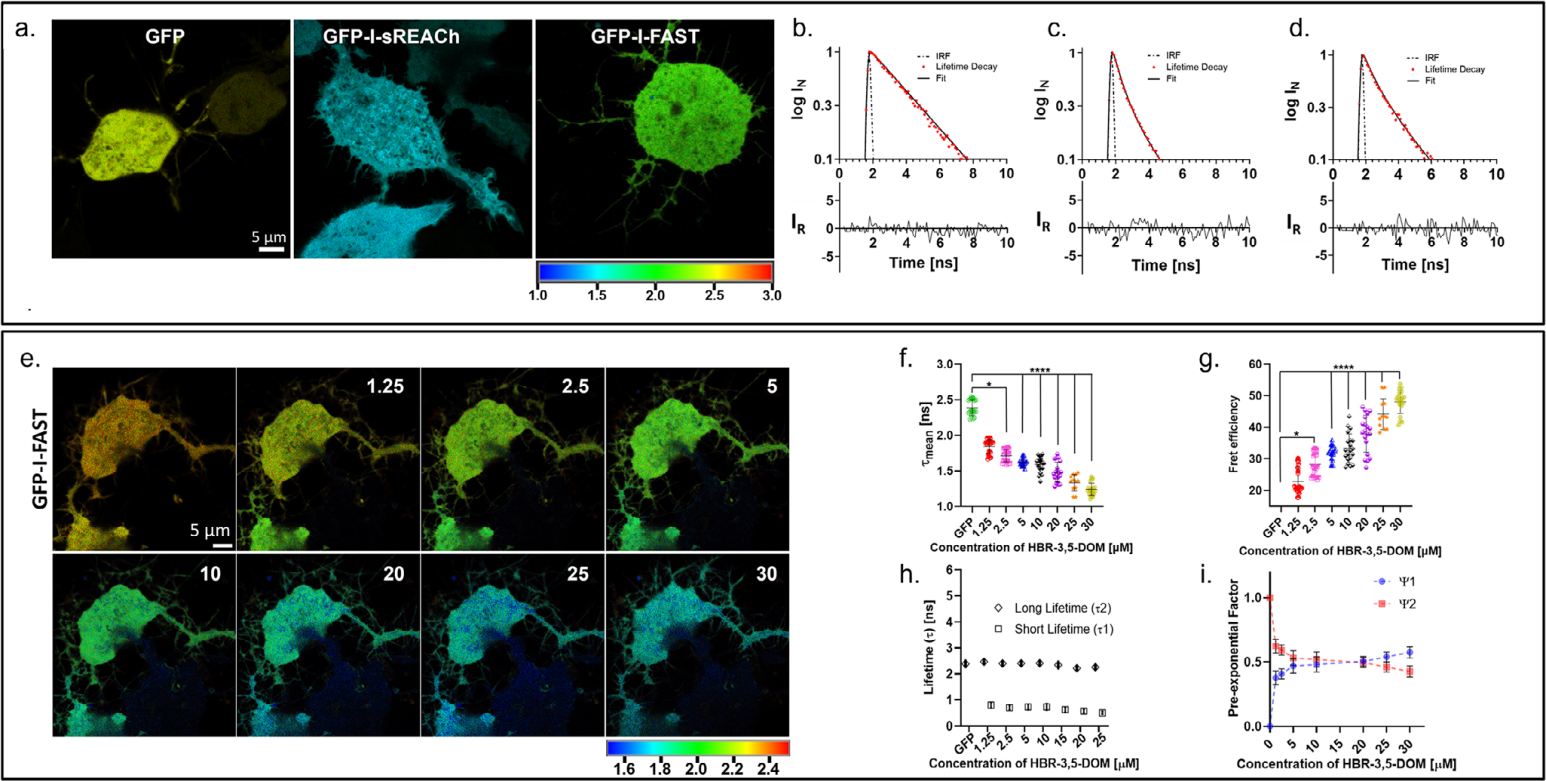
Chemically Controlled, Reversible FRET via Fluorogen‐Activated FAST. (a) FLIM images of Neuro‐2a cells expressing GFP alone (left), GFP‐I‐sREACh (middle, positive control), and GFP‐I‐FAST in the presence of 10 µM HBR‐3,5‐DOM (right). FLIM lifetimes are represented in pseudocolour (1.0–3.0 ns). (b‐d) Fluorescence decay analysis: GFP control showed a monoexponential decay (no FRET, negative control; left). GFP‐I‐sREACh displayed a biexponential decay (efficient FRET, positive control; middle). GFP‐I‐FAST also exhibited a biexponential decay, comparable to GFP‐I‐sREACh, indicating efficient FRET between GFP and fluorogen‐activated FAST (right). Data represent mean ± s.e.m. from ≥3 independent experiments (≥20 cells per condition). Scale bar: 5 µm. Fluorescence lifetime imaging microscopy (FLIM) of Neuro‐2a cells expressing GFP‐I‐FAST showed progressive reduction in donor lifetime with increasing concentrations of HBR‐3,5‐DOM (0–30 µm). Pseudocolour FLIM images showed a gradual shift from yellow/green to cyan/blue, reflecting increased FRET efficiency upon fluorogen binding. FLIM images are represented in a pseudocolor scalebar range 1.5–2.5 ns. (e) FLIM images of cells after sequential addition of HBR‐3,5‐DOM (0, 1.25, 2.5, 5, 10, 20, 25, and 30 µM). (f) Mean donor lifetime (τ) of GFP‐I‐FAST plotted against HBR‐3,5‐DOM concentration. A significant decrease in τ was observed from 2.5 µM onward, indicating enhanced FRET. (g) FRET efficiency increased in a concentration‐dependent manner, consistent with enhanced energy transfer upon fluorogen binding. (h) Long (τ_2_) and short (τ_1_) lifetime components remained relatively stable across concentrations, (i) Pre‐exponential factors Ψ_1_ (blue) and Ψ_2_ (red) showed opposite trends: Ψ_1_ increases and Ψ_2_ decreases, indicating a larger donor population undergoing FRET with increasing fluorogen. Data represent mean ± s.e.m. from ≥3 independent experiments (≥20 cells per condition). Scale bar: 5 µm.

Green fluorescent protein (GFP) remains one of the most widely used donor fluorophores in fluorescence lifetime–based Förster Resonance Energy Transfer (FRET) studies due to its excellent photophysical stability, well‐characterized quantum yield, and compatibility with live‐cell imaging. Its robust folding and expression in mammalian cells establish GFP as a benchmark for dynamic protein‐protein interaction assays. Among established donor‐acceptor pairs, GFP‐sREACh is one of the most efficient, with GFP as the donor and sREACh as a dark acceptor [35]. The high spectral overlap between GFP emission and sREACh absorption yields strong quenching and pronounced donor lifetime shortening, enabling high‐contrast FRET measurements in cellular environments (Figure 4a). To explore alternative, chemically tunable, and reversible acceptors, we evaluated the Fluorescence‐Activating and Absorption‐Shifting Tag (FAST) in combination with its cognate fluorogens, HBR‐3,5‐DM and HBR‐3,5‐DOM. Unlike fixed chromophore‐based acceptors, FAST allows dynamic control of fluorescence activation through reversible, non‐covalent binding of small‐molecule fluorogens, enabling modulation of fluorescence properties by ligand chemistry and cellular environment. Despite similar decay kinetics (Figure 3), spectral analyses revealed distinct excitation–emission profiles: HBR‐3,5‐DM displayed substantial spectral overlap with GFP emission, making optical separation of donor and acceptor signals during co‐expression difficult, whereas HBR‐3,5‐DOM showed a pronounced red shift, minimizing spectral bleed‐through and making it a better candidate for FRET with GFP (Figure 3c–f). Normalized absorption and emission spectra of GFP and the three FAST–fluorogen complexes, including the overlap between GFP emission and HBR‐3,5‐DOM absorption that underlies the FRET pairing, are provided in Figure S8 and are consistent with previously reported spectra for these fluorogens [23, 24] (Figure S8).

To assess FRET efficiency, we designed a GFP–FAST fusion construct (GFP‐I‐FAST) linked by a flexible Gly‐Ser‐rich peptide and compared it with a benchmark GFP‐sREACh fusion (GFP‐I‐sREACh) of similar linker length. In Neuro‐2a cells expressing GFP alone, fluorescence lifetime imaging microscopy (FLIM) revealed a monoexponential decay with a mean lifetime of 2.45 ± 0.02 ns, representing unquenched donor emission (Figure 4a,b, Figure S3a–d). GFP‐I‐sREACh exhibited strong donor quenching, reducing the mean lifetime to 1.06 ± 0.03 ns with biexponential decay components with independent fractional contributions (*τ*_1_ = 0.52 ± 0.04 ns, *τ*_2_ = 2.0 ± 0.05 ns; *Ψ*_1_ = 0.65 ± 0.02, *Ψ*_2_ = 0.34 ± 0.02), indicative of efficient FRET and partial heterogeneity in donor‐acceptor configurations (Figure 4a,c). In GFP‐I‐FAST cells, FRET‐mediated quenching occurred upon addition of 10 µM HBR‐3,5‐DOM. The donor lifetime decreased to 1.54 ± 0.09 ns, with biexponential decay components (*τ*_1_ = 0.73 ± 0.12 ns, *τ*_2_ = 2.41 ± 0.08 ns; *Ψ*_1_ = 0.48 ± 0.06, *Ψ*_2_ = 0.52 ± 0.06), reflecting two GFP populations; one engaged in FRET through fluorogen‐bound FAST and another unbound and unquenched (Figure 4a,d). This highlights how fluorogen binding governs FRET state occupancy which cannot be modelled with a monoexponential decay (Figure S5). Comparative analysis with GFP‐I‐sREACh underscores FAST‐fluorogen's utility as a tunable FRET acceptor with slightly lower maximal efficiency, but with chemical controllability advantageous for live‐cell imaging.

A key strength of the GFP‐FAST system lies in its intrinsic baseline controls. Because the fluorogen binds non‐covalently, the same cells can serve as their own controls: in the absence of ligand, GFP‐I‐FAST exhibits a monoexponential decay (τ ≈ 2.38 ± 0.11 ns) indistinguishable from GFP alone (Figure S6a, left), confirming negligible alteration from cells expressing GFP alone. Upon addition of 10 µM HBR‐3,5‐DOM, the donor lifetime decreases to 1.59 ± 0.11 ns, with biexponential decay revealing FRET‐active and unbound populations (Figure S6a, middle,b). Strikingly, washing out the fluorogen fully restores the donor lifetime (2.40 ± 0.10 ns) and monoexponential decay (Figure S6a right,c), demonstrating reversible, chemically gated FRET controlled by ligand association–dissociation rather than irreversible photophysical changes. We further examined fluorogen concentration dependence from 1.25–30 µm HBR‐3,5‐DOM (Figure 4e). Donor lifetime decreased gradually with increasing ligand, plateauing at 1.23 ± 0.09 ns at 30 µM, while calculated FRET efficiency increased from 22.85 ± 4.1% to 48.06 ± 3.7% (Figure 4f,g). Although τ_1_ and τ_2_ remained constant across concentrations, their pre‐exponential factors shifted: Ψ_1_ (short‐lifetime, FRET‐active) increased with ligand, and Ψ_2_ (long‐lifetime, unquenched) decreased, reflecting a concentration‐dependent redistribution of GFP from unbound to FRET‐engaged states (Figure 4h,i). This indicates that energy transfer efficiency is dictated primarily by the fraction of fluorogen‐bound FAST rather than conformational changes in the fusion protein, highlighting the intrinsic, self‐contained control of donor states. Collectively, these results establish GFP‐I‐FAST‐HBR‐3,5‐DOM as a versatile, dynamically controllable FRET platform. Unlike classical dark or permanently fluorescent acceptors, FAST enables optical modulation of FRET via external chemical inputs while maintaining intrinsic baseline controls, transforming energy transfer into a reversible, switchable signal.

### 2.4 Fluorogen‐Gated GFP–FAST as a Reversible Biosensor of Protein Conformational States

**FIGURE 5:**
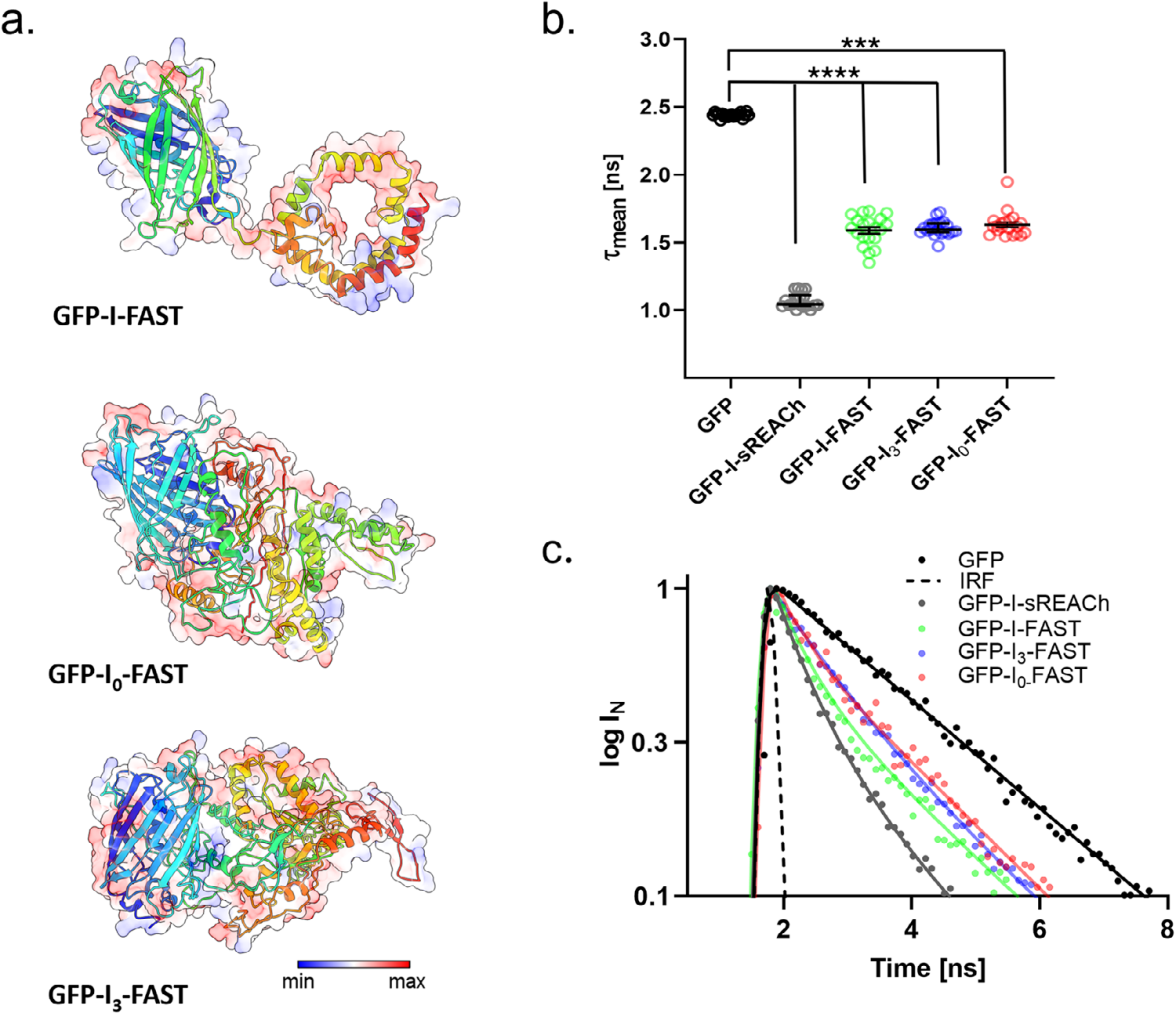
Mapping the C‐Terminal Supramodular Organization of MAGUKs Using Ligand‐Gated FLIM‐FRET. (a) Structural models of representative GFP fusion constructs GFP‐I‐FAST, GFP‐I_0_‐FAST, and GFP‐I_3_‐FAST showing different spatial orientations between GFP (yellow) and FAST (magenta). (b) Mean fluorescence lifetimes (τ) of GFP in Neuro‐2a cells expressing the indicated constructs in the presence of 10 µM HBR‐3,5‐DOM. GFP control (black) displayed a single long lifetime. A significant decrease in τ was observed in GFP‐I‐sREACh (grey), GFP‐I‐FAST (green), GFP‐I_0_‐FAST (red), and GFP‐I_3_‐FAST (blue), indicating efficient FRET. (c) Representative fluorescence lifetime decay curves for GFP and the GFP‐FAST fusion constructs, showing accelerated decay kinetics in FRET‐active constructs relative to GFP alone. These results confirmed that FAST, upon fluorogen binding, acts as an efficient acceptor for GFP in multiple fusion configurations. Data represent mean ± s.e.m. from at least three independent experiments (≥20 cells per condition).

To assess the broader utility of the GFP‐FAST system as a genetically encoded, reversible FRET biosensor, we engineered a set of intramolecular constructs that bridge GFP and FAST through defined structural modules. This design allows energy transfer to reflect the relative orientation and distance between donor and acceptor, thereby providing a readout of conformational states within multi‐domain proteins. We focused on the Discs large (DLG) subfamily of membrane‐associated guanylate kinases (MAGUKs), which includes PSD95, SAP97, SAP102, and PSD93 which are key scaffolding proteins in neuronal synapses and epithelial junctions. These proteins share a conserved modular architecture composed of three PDZ domains, an SH3 domain, and a guanylate kinase (GUK) domain, interconnected by flexible linkers and intrinsically disordered segments. Among these, the C‐terminal SH3–HOOK–GUK region plays a pivotal role in allosteric regulation and intramolecular communication. The HOOK region, which lies between SH3 and GUK, varies in sequence and flexibility across MAGUK family members and has been classified into isoforms I_0_ (PSD95) and I_3_ (SAP97) based on linker heterogeneity. This variation is thought to modulate the noncanonical SH3–GUK interaction and, consequently, the overall conformation and functional dynamics of the protein. To probe these conformational differences, we generated two FRET‐based constructs: GFP–I_0_–FAST and GFP–I_3_–FAST. Structural models of these fusion proteins, generated using the I‐TASSER prediction suite (Figure 5a), revealed distinct spatial arrangements of the donor and acceptor moieties, reflecting closed conformation in both I_0_ and I_3_ linkers. Fluorescence lifetime imaging microscopy (FLIM) was employed to quantify donor lifetimes and assess energy transfer efficiency under identical imaging conditions (Figure 5b). GFP control exhibited a monoexponential decay with a lifetime of 2.44 ± 0.02 ns, serving as the unquenched donor reference. In contrast, all fusion constructs incorporating FAST or sREACh displayed substantial reduction in mean donor lifetime, confirming efficient intramolecular FRET. The measured donor lifetimes were 1.06 ± 0.06 ns for GFP–I–sREACh, 1.59 ± 0.10 ns for GFP–I–FAST, 1.63 ± 0.09 ns for GFP–I_0_–FAST, and 1.60 ± 0.05 ns for GFP–I_3_–FAST. The corresponding FRET efficiencies, calculated from lifetime quenching relative to the GFP control, were 56.1%, 34.8%, 34.2%, and 33.1%, respectively (Figure 5c). While sREACh, as expected, provided the highest energy transfer efficiency due to its strong spectral overlap and high extinction coefficient, the FAST‐based constructs demonstrated remarkably consistent and reproducible FRET upon fluorogen activation. The observed lifetimes for GFP–I_0_–FAST (*τ*_1_ = 0.83 ± 0.11 ns; *τ*_2_ = 2.2 ± 0.09 ns) and GFP–I_3_–FAST (*τ*_1_ = 0.76 ± 0.11 ns; *τ*_2_ = 2.3 ± 0.06 ns) followed a biexponential decay pattern with nearly equal amplitude contributions (*Ψ*_1_ = 0.44 ± 0.04, *Ψ*_2_ = 0.56 ± 0.04 for I_0_; *Ψ*_1_ = 0.45 ± 0.03, *Ψ*_2_ = 0.55 ± 0.03 for I_3_). This dual‐component decay reflects a mixture of FRET‐engaged and non‐FRET donor populations, consistent with partial activation of FAST by fluorogen and conformational heterogeneity within the SH3–HOOK–GUK module.

Importantly, the close similarity in both lifetimes and FRET efficiencies between GFP–I_0_–FAST and GFP–I_3_–FAST suggested that local structural differences in the hook region do not drastically alter the mean donor–acceptor separation or orientation under resting conditions. This finding indicates that the FAST‐based FRET pair is sufficiently sensitive to detect domain rearrangements but tolerant of flexible linkers—properties highly desirable in biosensor design (Figure S7). The consistency of these results also supports the mechanistic interpretation that the principal determinant of energy transfer is the interdomain geometry rather than ligand‐binding kinetics or fluorogen exchange. Beyond confirming efficient energy transfer, the GFP–FAST system introduces two key advantages over conventional FRET acceptors. First, fluorogen‐dependent activation permits temporal control where researchers can initiate or terminate FRET by simple addition or removal of the small molecule ligand, allowing dynamic monitoring of conformational transitions in live cells. Second, the use of a genetically encoded donor and a chemically gated acceptor minimize spectral bleed‐through and may help limit phototoxicity [26], while maintaining a robust signal‐to‐noise ratio. Together, these results establish the GFP–FAST combination as a powerful intramolecular FRET platform capable of resolving domain level conformational states in multidomain proteins. In the context of MAGUK scaffolds, this system provides a direct optical handle to interrogate the equilibrium between “open” and “closed” SH3–GUK configurations and to assess how these configurations are influenced by local binding events or post‐translational modifications. Because the FAST fluorogen can be reversibly applied, such conformational equilibria can now be studied in real time, within the same cellular population, without the need for multiple genetic constructs or differential labelling. In summary, the GFP–FAST pair extends the capabilities of traditional FRET sensors by introducing a chemically controllable, reversible acceptor system that faithfully reports on nanoscale structural dynamics. When coupled with advanced FLIM analysis, this platform offers a quantitative and flexible approach for mapping protein conformations in living cells—bridging the precision of structural biology with the temporal resolution of optical biosensing.

## 3 Discussion

The Fluorescence‐Activating and Absorption‐Shifting Tag FAST represents a chemobiological hybrid system in which optical output arises from reversible association between a genetically encoded scaffold and a synthetic ligand [21, 22, 36]. Unlike classical fluorescent proteins that embed a covalently matured chromophore, FAST generates fluorescence through non‐covalent fluorogen confinement, rendering emission chemically gated, reversible, and programmable. In this framework, the emissive state is not pre‐installed but emerges from ligand‐induced remodelling of the protein by transient association. By integrating X‐ray crystallography, computational ensemble analysis, FLIM, and ligand‐controlled FRET, a quantitative structural‐photophysical relationship is established linking ligand geometry to excited state energy surfaces, radiative decay rates, and conformational ensemble partitioning in living cells. The crystal structure of FAST bound to HBR‐3,5‐DM reveals a planar, interlocked chromophore stabilized by π–π stacking, hydrogen bonding, and confinement within a hydrophobic interfacial pocket formed by a domain‐swapped dimer. In the apo state, these observations agree with prior work showing that N‐terminal residues occupy the cleft and stabilize a self‐filled ground‐state minimum. Ligand binding then displaces these residues without large‐scale domain rearrangement, redefining steric and electrostatic boundary conditions around the chromophore, consistent with earlier evidence that ligand engagement orders the FAST scaffold and stabilizes the bound state [27, 29]. This localized remodelling likely restricts torsional motion along the methine bridge, a major coordinate for ultrafast internal conversion in free HBR‐like fluorogens, thereby elevating torsional barriers and suppressing non‐radiative decay. The resulting single, well‐defined minimum on the FAST::HBR‐3,5‐DM energy surface and monoexponential fluorescence decay indicate a predominant resolvable emissive state representing a uniform radiative decay across the bound population. In contrast, flexible fluorogens such as HMBR resist crystallographic trapping and adopt multiple docking geometries. Computational alignment reveals retained rotational and translational freedom within the binding pocket, producing several quasi‐stable excited‐state conformations separated by shallow barriers. The FAST::HMBR energy surface exhibits multiple minima. In an ensemble these conformations are not uniformly populated; one conformation dominates while the second‐most populated state contributes significantly. These coexisting configurations access alternative internal conversion pathways, giving rise to biexponential decay kinetics because of multiple radiative decay contributions from dominant moieties in FAST::HMBR labelled cells. Notably, the lifetime heterogeneity is independent of ligand concentration, indicating that the observed photophysics is governed by ensemble partitioning of conformations within the bound state rather than by exchange kinetics. Similarly, the FAST::HBR‐3,5‐DOM energy surface also shows multiple minima. However, unlike FAST::HMBR, a single conformation dominates the bound population, and contributions from other states are minimal, resulting in monoexponential lifetime decay. For HBR‐3,5‐DOM, the dominant docked clusters are nearly identical in both energy and binding geometry, resulting in indistinguishable lifetimes that collapse into a single predominant resolvable emissive state. In contrast, the major HMBR clusters are more distinct structurally and energetically, giving rise to resolvable lifetime populations and a multiexponential decay. These observations show that chromophore rigidity and spatial confinement shape the topology and dimensionality of the excited‐state landscape. Planar ligands restrict the system to a single emissive basin, while flexible ligands generate rugged surfaces sampled stochastically during relaxation, in line with prior structure‐based and fluorogen‐engineering work on FAST [27, 30, 32]. Water molecules bridging FAST‐ligand interaction may further modulate the energy landscape by influencing local dielectric stabilization and charge redistribution in the excited state.

Subcellular FLIM of FAST::HMBR and FAST::HBR‐3,5‐DOM reveal that mitochondrial targeting shortens lifetimes, Lyn‐mediated membrane anchoring prolongs them, and microtubule association preserves cytosolic‐like kinetics with single‐exponential character. These shifts are consistent with environmental modulation of non‐radiative decay through polarity, viscosity, and electrostatic effects [21, 30]. A key question was whether the excited‐state dynamics inferred from crystal structures and soluble FAST would remain valid in fusion proteins, where the N‐ and C‐termini are constrained and not free to assemble as tandem dimers. Although fluorescence lifetimes in fusion constructs changed in some compartments such as mitochondria and membrane, others showed little difference, including microtubules and volumetric labelling. These differences appear to arise largely from the intracellular microenvironment rather than from changes in formation of the FAST–ligand complex itself. This supports the view that the binding interface directly governs the stochastic assembly processes that generate the fluorescent moieties. Together, FAST acts as a nanoscale lifetime reporter, translating microenvironmental fluctuations into quantifiable lifetime changes [8, 9]. Beyond photophysical characterization, the conditional nature of fluorogen binding enables a methodological advance: reversible, chemically gated FRET [5, 21]. Using HBR‐3,5‐DOM as a spectrally optimized acceptor, GFP–FAST constructs exhibit ligand‐dependent donor lifetime shortening that is reversible upon washout. Time‐resolved analysis shows that FRET modulation arises from redistribution of FRET‐active and inactive donor populations rather than from alteration of intrinsic donor decay constants. This chemical gating introduces an orthogonal control parameter: ligand concentration that modulates energy transfer independent of expression level or protein architecture, allowing each cell to serve as its own internal control [21]. Such reversibility overcomes limitations of constitutive fluorescent acceptors and strengthens quantitative FLIM‐FRET as a method for probing dynamic protein organization [1, 4, 37].

Application to MAGUK scaffolds, including PSD95 and SAP97, illustrates the power of this approach. Intramolecular GFP–FAST constructs spanning PDZ–SH3–GK supramodular assemblies report ligand‐dependent lifetime components that are most naturally interpreted in the context of a conformationally coupled MAGUK core rather than isolated domains [14, 15, 16, 17]. This interpretation is also consistent with prior evidence that PSD95 samples regulated super tertiary states and that SAP97 can adopt both compact and elongated forms [38, 39, 40, 41]. Because acceptor activation is conditional, ligand washout validates proximity derived FRET signals and helps exclude static spectral artifacts, extending earlier FLIM‐FRET and related proximity‐based approaches to study PSD95 organization in cells [18, 19, 20, 42]. The GFP–FAST pair thus operates as a reversible optical ruler, mapping conformational equilibria as emergent properties of ligand‐defined excited‐state landscapes. Collectively, these findings establish a paradigm linking ligand chemistry, binding geometry, chromophore rigidity, solvation structure, and excited‐state topology to fluorescence lifetime and FRET behavior, extending the design logic emerging from recent FAST structure and fluorogen studies [21, 27, 29, 30]. FAST functions not merely as a conditional fluorophore but as a chemically programmable module to report excited‐state reactions in which synthetic ligand design directly modulates the topology of dipole‐dipole transitions and decay pathways. By coupling structural precision with stochastic excited‐state sampling and reversible energy transfer control, this work defines design principles for engineering ligand‐regulated fluorescence systems and expands the methodological repertoire for quantitative structural biology in living cells. This framework positions FAST as a next‐generation platform at the intersection of chemistry and cell biology. It demonstrates that excited‐state energy landscapes, ensemble partitioning, and environmental responsiveness can all be tuned via ligand structure and its engagement, offering predictive control over radiative versus non‐radiative ratios, lifetime distributions, and energy transfer efficiency. The combination of chemically tunable fluorescence, structural inferences, and reversible FRET establishes FAST as both a mechanistic probe of molecular architecture and a blueprint for rationally designing future photophysical systems to quantify conformational and interaction maps of biomolecules in living systems.

The advantage of choosing a fluorogen‐activated acceptor over a conventional fluorescent‐protein or dark acceptor is not raw transfer efficiency. The dark acceptor sREACh produced a larger maximal lifetime quenching than fluorogen‐activated FAST, but the strength of FAST lies in control, tunability, and internal calibration. First, because activation is reversible and ligand‐gated, energy transfer can be switched on and off and titrated within a single living sample, so that each cell provides its own donor‐only baseline and proximity‐derived signals can be validated by ligand washout, eliminating the need for separate control populations or paired constructs. Second, the photophysical output is programmable through ligand chemistry rather than protein re‐engineering: substituting one fluorogen for another retunes spectra, lifetime multiplicity, and FRET efficiency, as demonstrated across the HMBR/HBR‐3,5‐DM/HBR‐3,5‐DOM series. Third, the fluorogenic, dark‐until‐bound nature of the acceptor suppresses background and, together with the small size of the tag (∼14 kDa, versus the ∼27 kDa β‐barrel of fluorescent proteins), may contribute to reduced phototoxicity and steric perturbation of the host protein [26]. Finally, because the donor lifetime is governed by the fraction of fluorogen‐bound acceptor and is largely insensitive to ligand concentration in the saturated regime, the readout is quantitative and robust. The central conclusion of this work is that the identity of a non‐covalent ligand can program the excited‐state landscape of a fluorogen‐protein complex, dictating whether the bound population occupies a single emissive basin or a rugged surface of comparably populated states, and that this landscape can be read out directly as fluorescence‐lifetime multiplicity and as tunable, reversible FRET. These principles are not specific to FAST. The same logic, relating the convergence and separation of bound conformational sub‐states to lifetime homogeneity, using graded ligand substitution to tune spectra and excited‐state topology, and exploiting reversible binding to build internal controls, would extend to other fluorogen‐activating proteins (for example the Y‐FAST, pFAST, and frFAST variants). It could also be applied to fluorogen‐activating peptide and malachite‐green systems, to RNA aptamer–fluorogen pairs such as Spinach, Mango, and Pepper, and more broadly to self‐labelling tags charged with environment‐sensitive dyes [43]. In each of these systems, choosing or engineering ligands for conformational rigidity and for spectral complementarity with a donor offers a rational route to predictable lifetime behavior and to chemically gated, reversible energy‐transfer reporters.

## 4 Materials and Methods

### 4.1 Mammalian Cell Culture

Neuroblastoma cells (Neuro‐2a, ATCC CCL‐131) were cultured in DMEM supplemented with GlutaMAX, 1% fetal bovine serum (FBS), and 1% penicillin‐streptomycin at 37°C in a humidified 5% CO_2_ atmosphere. For imaging, cells were seeded on 18 mm #1.5 glass coverslips (VWR) and transiently transfected with plasmid DNA using Lipofectamine 3000 (Thermo Fisher Scientific) according to the manufacturer's instructions. Sixteen hours post‐transfection, coverslips were mounted in a custom Ludin chamber (Life Imaging Services) filled with extracellular solution (ECS: 120 mM NaCl, 3 mM KCl, 2 mM CaCl_2_·2H_2_O, 1 mM MgCl_2_·6H_2_O, 10 mM D‐glucose, 10 mM HEPES, pH 7.4). Fluorogens were added directly to the chamber to achieve desired final concentrations for live‐cell imaging [26].

### 4.2 Plasmids and Fluorogens

Constructs included Ensconsin‐FAST, Lyn11‐FAST, Mito‐FAST [22], GFP–FAST variants (GFP‐I‐FAST, GFP‐I_0_‐FAST, GFP‐I_3_‐FAST), and GFP‐I‐sREACh controls (mGFP‐10‐sREACh‐N3‐Addgene plasmid #21947) [35, 44]. Fluorogens used were HMBR, HBR‐3,5‐DM, and HBR‐3,5‐DOM, which are commercially available (e.g., The Twinkle Factory) and, in this study, were synthesized following published procedures and in part provided by the laboratory of A. Gautier. HMBR was synthesized following Plamont et al., 2016, and the HBR derivatives were prepared as described in Li et al., 2017. Stocks were maintained at 10 mM in DMSO and diluted in PBS immediately prior to experiments.

### 4.3 Absorption and Emission Spectroscopy

Absorption and emission spectra of purified GFP (15 µM / 150 nM) and purified FAST::fluorogen (50::50 µM / 500:500 nM) complexes were recorded. For absorption spectra, purified FAST (50 µM) was incubated with each fluorogen (HMBR, HBR‐3,5‐DM, or HBR‐3,5‐DOM; 50 µM) in 15 mM Tris‐HCl (pH 7.4) and 150 mM NaCl, whereas for emission spectra, 500 nM of ligand and fluorogen were used for complex formation for the recordings. Absorption spectra were recorded on a Jasco‐V‐530‐UV–visible spectrophotometer with a 1‐cm quartz cuvette at room temperature. Spectra were collected from 300 to 700 nm. Emission spectra were recorded on a FluoroMax‐3 spectrofluorimeter with each FAST::fluorogen complex being excited at its absorption maximum. GFP emission was recorded under 488 nm excitation. Spectra were normalized to their respective maxima for display and for evaluation of donor–acceptor spectral overlap. Background spectra from buffer and free fluorogen were subtracted before analysis.

### 4.4 Molecular Cloning

- **FAST‐C1**: FAST coding sequence was amplified from Mito‐FAST and ligated into mEos3.2‐C1 Plasmid #54550 [45] via NheI/SalI digestion.
- **GFP‐I‐FAST**: FAST replaced sREACh in GFP‐I‐sREACh via BamHI/NotI digestion. Positive clones were sequence‐verified. Linker I amino acid sequence‐VDGTAGPGS.
- **GFP‐I_0_/I_3_‐FAST**: SH3–GUK fragments were amplified from hDLG1 cDNA and inserted into GFP‐I‐FAST vector using Gibson Assembly (New England Biolabs, Cat. No. E2611). Clones were validated by colony PCR and Sanger sequencing.
- **pET28a‐FAST**: FAST was subcloned into pET‐28a (BamHI/HindIII sites) for bacterial expression. Single‐point mutations were corrected via site‐directed mutagenesis; sequences were verified by Sanger sequencing Table S5.

### 4.5 Protein Expression and Purification

- *Protein expression*: *Escherichia coli* BL21(DE3) cells were transformed with pET‐28a‐FAST. Cultures were grown at 37°C until OD_600_ reached 0.8, followed by induction with 0.6 mM IPTG (Thermo Fisher Scientific) overnight. Cells were harvested by centrifugation at 4000 × g for 10 min and stored at −80°C. Pellets were resuspended in 10 mM Tris‐HCl (pH 7.4), 150 mM NaCl, supplemented with protease inhibitors (Roche), and lysed by sonication (Vibra Cell, Sonics).
- *Purification*: Lysates were clarified at 30 000 × g for 1 h, and the supernatant was incubated with Ni^2^^+^‐NTA agarose resin (Qiagen). Beads were washed with 4 column volumes of 10 mM Tris‐HCl (pH 7.4), 150 mM NaCl, 30 mM imidazole, and proteins were eluted in 10 mM Tris‐HCl (pH 7.4), 150 mM NaCl, 250 mM imidazole. Eluates were concentrated to 500 µL using Amicon Ultra centrifugal filters (3 kDa cutoff) and clarified by ultracentrifugation at 35 000 × g for 30 min at 4°C to remove aggregates.
- *Size‐exclusion chromatography*: Purified proteins were injected onto a Superdex 75 column pre‐equilibrated with 10 mM Tris‐HCl (pH 7.4), 150 mM NaCl using an ÄKTA pure system with Unicorn 7.3 software. Protein purity was confirmed by SDS‐PAGE, and concentrations were determined from A_280_ measurements and corresponding extinction coefficients (NanoDrop, Thermo Scientific).
- *SDS‐PAGE*: Samples (15 µg protein in 1× Laemmli buffer) were resolved on 15% resolving / 4% stacking gels (Bio‐Rad) and run in Tris‐Glycine‐SDS running buffer. Gels were stained with Coomassie Blue, destained with methanol:acetic acid:water (4:1:5), and imaged using a Gel Doc EZ (Bio‐Rad) [11].

### 4.6 Crystallization and Structure Determination

Purified FAST protein was concentrated to 8–12 mg mL^−^^1^ and incubated with 1 mM HBR‐3,5‐DM for 2 h prior to crystallization. Apo and ligand‐bound FAST were crystallized using hanging‐drop vapor diffusion at 20°C. Initial conditions were based on 0.2 M magnesium formate, 20% (w/v) PEG 3350, pH 5.5, with grid screening for pH 5.3–5.8 and PEG 14–26% (w/v). Crystals grew primarily at ≥20% PEG; ligand‐bound FAST required microseeding using crushed apo crystals to improve growth. Diffraction screening was performed in‐house; high‐resolution data were collected at the Elettra XRD2 beamline. Apo FAST diffracted to 2.9 Å, ligand‐bound FAST to 1.6 Å. Data processing was performed using CCP4i2: integration with MOSFLM, scaling/merging with AIMLESS. Phases were obtained by molecular replacement with PHASER using apo FAST (PDB: 7AV6) as the search model. Ligands and waters were fitted in Fo‐Fc omit maps using COOT and refined iteratively with Phenix.refine (PHENIX suite) [29, 46].

### 4.7 Molecular Docking of Fluorogen‐FAST Interactions

The 2D representations of the fluorogens were made using RDKit: Open‐Source Cheminformatics Software (rdkit/rdkit: 2026_03_3 (Q1 2026) Release | Zenodo). The HBR‐3,5‐DM‐bound FAST crystal structure was used for in silico docking analyses with HBR‐3,5‐DOM and HMBR. SMILES representations (PubChem: CID 118628175 and CID 3003840) [47] were converted to MOL2 format using OpenBabel 2.4.1 and prepared in PDBQT format via MGLTools 1.5.6 [48] (ligand preparation: rotatable bonds, centre identification) [49]. The FAST receptor (PDB) with HBR‐3,5‐DM removed was similarly converted using MGLTools (receptor preparation). Grid maps were centred on the original HBR‐3,5‐DM coordinates (spacing = 0.375 Å) and were made to cover interacting residues. Docking was performed in AutoDock 4.2.6 using a genetic algorithm (population size = 150; max evaluations = 25 000 000; max generations = 27 000) [50], generating 50 poses per ligand. Docked complexes were clustered, and the lowest energy pose from each cluster was selected. Interaction visualizations and comparative analyses were performed using LigPlot+ v2.3 [51].

### 4.8 Fluorescence Lifetime Imaging Microscopy (FLIM)

Live‐cell fluorescence lifetime imaging (FLIM) was performed on a Leica TCS SP8 FALCON confocal system (Leica Microsystems, Wetzlar, Germany), providing high temporal resolution and photon efficiency for time‐domain lifetime measurements. A 63×/1.4 NA oil‐immersion objective was used to achieve high spatial resolution and optimal light collection. Hybrid single‐molecule detection (HyD SMD) photon‐counting detectors enabled precise lifetime measurements with minimal background noise. Excitation was delivered by a pulsed white light laser (WLL) operating at 40 MHz, allowing simultaneous excitation of multiple fluorophores. Excitation wavelengths were selected to match the absorption maxima of each fluorogen: 488 nm for HMBR and HBR‐3,5‐DM, and 518 nm for HBR‐3,5‐DOM. Emission signals were spectrally separated using tunable detection windows: 520–560 nm for HMBR, 520–590 nm for HBR‐3,5‐DM, and 580–650 nm for HBR‐3,5‐DOM [23]. For FRET measurements, GFP was selectively excited at 488 nm, and donor emission was collected between 498 and 550 nm. The 488 nm excitation intensity was selected to minimize direct excitation of HBR‐3,5‐DOM. The acceptor channel was recorded when required to confirm FRET and rule out spectral bleed‐through. Laser intensity was optimized to maintain a photon detection rate of 0.02–0.2 photons per pulse, minimizing pile‐up artifacts and ensuring accurate lifetime determination. Detector gain and offset were adjusted to avoid saturation and maximize dynamic range. Images were acquired using bidirectional scanning at 400 Hz with 512 × 512 pixels, balancing acquisition speed and photon collection efficiency. For each field of view, acquisition continued until a minimum of 500 photons per pixel were collected, providing sufficient statistics for multi‐exponential lifetime fitting. Instrument response function (IRF) calibration was performed using a standard lifetime reference [33]. All imaging was conducted under controlled environmental conditions to maintain cell viability.

### 4.9 FLIM Data Processing and Analysis

Fluorescence decay curves were analysed using the LASX FALCON software (Leica Microsystems). Decays were fitted using an *n*‐exponential reconvolution model with the pre‐calculated IRF. Circular regions of interest (ROIs) were manually defined and only fits with reduced chi‐square (χ^2^) ≤ 1.2 were included for further analysis.

Fluorescence decay for predominant resolvable emissive states were modeled as:

$$N\left(t\right)=\sum_{i=1}^{n}N_{0,i}\;e^{-k_{i}t}$$

where *N*(*t*) is the total intensity at time *t*, *N*_0,*i*_ is the initial amplitude of the *i*‐th component, *k_i_* =  1/τ_*i*_ is the decay constant, and *n* is the number of exponential components. Model improvement was accepted only if $\chi_{i=n}^{2}/\chi_{i=n+1}^{2}\geq 1.05$.

For FRET biosensors, donor decays were represented by a biexponential model:

$$I\left(t\right)=A_{1}\;e^{-t/\tau_{1}}+A_{2}e^{-t/\tau_{2}}$$

where τ_1_ corresponds to the unquenched donor lifetime, τ_2_ corresponds to the shortened lifetime in the presence of an acceptor, and *A*_1_and *A*_2_ represent the relative fractions of donor molecules unbound or engaged in FRET, respectively. Relative fractions of donor molecules were then normalized and presented as Pre‐exponential factors:

$$\;\psi_{1}=\frac{A_{1}}{A_{1}+A_{2}}\;,\;\psi_{2}=\frac{A_{2}}{A_{1}+A_{2}}$$

The amplitude‐weighted mean lifetime (τ_*amp*_) was calculated as:

$$\;\tau_{mean}=\psi_{1}\;\tau_{1}+\psi_{2}\tau_{2}$$

FRET efficiency (*E_i_*) was determined using:

$$\;E_{i}=1-\frac{\tau_{DA}}{\tau_{D}}$$

where τ_*D*_and τ_*DA*_denote donor lifetimes in the absence and presence of acceptor, respectively.

Lifetime data were acquired with a temporal resolution of 0.09 ns per channel, and differences below 0.20 ns were considered within the limits of experimental uncertainty based on the instrument's temporal sampling criteria. However, closely spaced states that may not be resolvable may fall below the temporal resolution of the measurement. This analysis enables a robust, concentration‐independent quantification of molecular interactions, local microenvironment alterations, or conformational states in live cells.

### 4.10 Statistical Analysis

Statistical analyses were performed using GraphPad Prism (v10). Normality of the datasets was assessed using the Shapiro–Wilk test. Parametric datasets were evaluated using a Brown–Forsythe test to determine significance across groups, whereas non‐parametric datasets were analysed using a Kruskal–Wallis test followed by appropriate post hoc comparisons. Statistical significance was defined as *p* < 0.05, *p < 0.01, **p < 0.005, and ***p < 0.0005. Data are presented as mean ± standard error of the mean (SEM) in figures, and as mean ± standard deviation (SD) or median ± interquartile range (IQR) in tables and other reporting, unless otherwise stated.

### 4.11 Image Rendering and Language Corrections

Schematic workflows for imaging and analysis were created using BioRender (paid subscription). Large language models (GPT‐4, Gemini 1/2) were used solely for language editing and sentence clarity, not for research content generation or data analysis. Illustrative image for graphical abstract was done with the aid of Claude Opus 4.8. The authors are fully responsible for the conceptual design, scientific rationale, results, and data interpretation.

## Author Contributions

N.S., M.J., and D.N. designed the research. N.S. performed all experiments unless otherwise noted and prepared the Neuro‐2a cell lines. N.S. and D.N. conducted data analysis. N.S. and A.S. performed FLIM imaging. N.S., A.H., and A.S. generated fusion constructs. N.S. purified and characterized the proteins. S.N. and N.S. carried out all crystallization experiments, and S.N. and A.P. performed crystal analysis. S.K.M. conducted in silico interaction analyses. N.S., S.K.M., S.N., A.P., and D.N. wrote the manuscript. All authors read, provided critical input, and approved the final version of the manuscript.

## Conflicts of Interest

D.N. is the CEO and Director of Research at eNLife Research Pvt. Ltd., India, a company involved in early diagnosis research for neurodegenerative diseases. This role does not constitute a conflict of interest with respect to the present study. All other authors declare no conflicts of interest.

## Supporting information




**Supporting File**: advs77693‐sup‐0001‐SuppMat.docx.

## Data Availability

The data that support the findings of this study are available on request from the corresponding author. The data are not publicly available due to privacy or ethical restrictions. The experimental X‐ray data and refined models for the Apo FAST and FAST bound to HBR‐3,5‐DM are deposited in the protein data bank (PDB) with the IDs 45SD and 45TX, respectively.
