# Supplementary material for: Reversible, Chemically Gated FRET via Ligand‐Activated Acceptors
Source: Adv Sci (Weinh). 2026 Sep 27:e77693. Online ahead of print. doi: 10.1002/advs.77693 (PMC13616411; doi:10.1002/advs.77693)
Supplement: Supplementary file 1 [file ADVS-9999-e77693-s001.docx]

**Reversible, Chemically Gated FRET via Ligand-Activated Acceptors**

Nivedita Singh^1^, Smruti Ranjan Nayak^2^, Sanjay Kumar Mohanty^1^, Aishwarya Hanumantharaju^1^, Amala Shaju^1^, Mini Jose^3^, Aravind Penmatsa^2^, Deepak Nair^1, #^

^1^Centre for Neuroscience, Indian Institute of Science, Bangalore 560012 (India).

^2^Molecular Biophysics Unit, Indian Institute of Science, Bangalore 560012 (India).

^3^Cell and Tissue Imaging Center-Light Microscopy, St Jude Children’s Research Hospital, Memphis, 38105 (USA).

^#^ Correspondence should be addressed to Deepak Nair, deepak@iisc.ac.in

**Supplementary Data**

**Supplementary Figure Legends**

**Supplementary Figure 1. Ligand Interaction Maps of FAST with HBR-3,5-DOM and HMBR.**

a) Ligplots depicting the interacting amino acid residues of FAST and atoms of the HBR-3,5-DOM in the respective binding mode. b) Ligplots depicting the interacting amino acid residues of FAST and atoms of the HMBR in the respective binding mode.

**Supplementary Figure 2. FLIM-Phasor Validation of Monoexponential Fluorophore Lifetimes of Rhodamine B and Coumarin 6.**

(a, d) FLIM images of Rhodamine B (a) and Coumarin 6 (d). FLIM images are represented in a pseudocolour scalebar range 1.0-3.0 ns. (b, e) Fluorescence intensity decays of Rhodamine B (b) and Coumarin 6 (e), acquired at 543 nm and 457 nm, respectively. Emission was collected in the 570–650 nm range for Rhodamine B and 470–540 nm for Coumarin 6. Both decays fit by a monoexponential decay model, indicating homogeneous fluorophore populations. Goodness of monoexponential decay is indicated by the residual fit shown below the intensity decays with mean lifetimes of 1.45 ± 0.06 ns and 2.45 ± 0.02 ns, respectively. (c, f) The phasor plot analysis of Rhodamine B (c) and Coumarin 6 (f), each showing phasor points localized on the universal semicircle, validating their monoexponential decay. Data represent mean ± SD. from at least three independent experiments (≥5 per condition). Scale bar: 5 μm.

**Supplementary Figure 3. Comparative FLIM Analysis of GFP and FAST–HMBR in Neuro-2a Cells.**

(a) Fluorescence intensity images (left) and FLIM images (right) of Neuro-2a cells expressing GFP. (e) Fluorescence intensity images (left) and FLIM images (right) of Neuro-2a cells expressing FAST labelled with HMBR. FLIM images are represented in a pseudocolour scalebar range of 1.0-3.0 ns. (b, f) FLIM histograms showing mean fluorescence lifetimes of 2.45 ± 0.02 ns for GFP (b) and 1.48 ± 0.09 ns for the FAST-HMBR complex (f). (d, h) Fluorescence intensity decay of GFP (d) and FAST-HMBR (h), both excited at 488 nm. The GFP decay was fitted using a monoexponential model, characteristic of a homogeneous population, while FAST-HMBR showed a biexponential decay profile, suggesting a mixed population of two FAST-HMBR complexes (c, g) Phasor plot analysis of GFP (c) and FAST-HMBR (g). The GFP phasor is confined to the universal semicircle, confirming monoexponential decay, while the FAST-HMBR phasor shifts inside the semicircle, consistent with a biexponential distribution. Data represent mean ± SD. from at least three independent experiments (≥10 cells per condition). Scale bar: 5 μm.

**Supplementary Figure 4. Ligand Concentration Has Minimal Impact on FAST–Fluorogen Excited-State Dynamics.**

Amplitude-weighted mean fluorescence lifetimes (τ) of FAST bound to different fluorogens were measured across a concentration range of 1.25 μM to 10 μM. Amplitude-weighted mean lifetime (τ) of the FAST-HMBR complex (a) FAST-HBR-3,5-DM complex (b) and FAST-HBR-3,5-DOM complex (c) with different concentrations of fluorogens. All the complexes showed non-significant change in lifetime (τ), indicating strong and consistent interaction with the FAST tag that is largely independent of fluorogen concentration. Data represent mean ± s.e.m. from at least three independent experiments (≥20 cells per condition).

**Supplementary Figure 5. Fluorescence Decay Analysis of GFP-Based FRET Constructs.**

(a) Monoexponential decay of GFP-I-sREACh. (b) The monoexponential model did not adequately fit the mean fluorescence decay of GFP-I-FAST and GFP-I-sREACh, supporting the occurrence of additional excited state reaction between GFP and its acceptors. Data represent mean ± s.e.m. from ≥3 independent experiments (≥20 cells per condition).

**Supplementary Figure 6. Reversible Fluorogen Labelling Enables Autonomous FRET Control with FAST.**

FAST enables a control-independent FRET. (a) FLIM images of Neuro-2a cells expressing GFP-I-FAST under three conditions: in the absence of HBR-3,5-DOM (left), after addition of 10 μM HBR-3,5-DOM (middle), and following fluorogen washout (right). A clear reduction in donor lifetime was observed upon fluorogen addition, which recovered after washout. FLIM images are represented in a pseudocolour scalebar range of 1.0-3.0 ns. (b) The donor lifetime (control) significantly decreased from ~2.38 ± 0.11 ns to ~1.59 ± 0.10 ns upon addition of HBR-3,5-DOM, confirming efficient FRET resulting from fluorogen binding to the FAST. The biexponential fluorescence intensity decay observed indicated the presence of both interacting (FRET) and non-interacting donor population. (c) Upon removal of the fluorogen, the donor lifetime reverted to monoexponential decay, indicating reversibility and the loss of FRET. FLIM images are represented in a pseudocolour scalebar range 1.0-3.0 ns. Data represent mean ± SD from at least three independent experiments (≥20 cells per condition). Scale bar: 5 μm.

**Supplementary Figure 7. Schematic representation of HBR-3,5-DOM binding to GFP-I-FAST and resulting FRET.**
The diagram illustrates that the same GFP-I-FAST construct can serve as both a control and a FRET pair when FAST is used as the acceptor. It depicts the mechanism of fluorogen binding and fluorescence energy transfer in the GFP-I-FAST fusion protein. **In the absence of fluorogen (top):** GFP (green, donor) and FAST (blue, acceptor) remain non-fluorescently coupled, resulting in no FRET and a normal GFP fluorescence lifetime. **Upon addition of HBR-3,5-DOM (middle):** The fluorogen binds specifically to FAST, forming a fluorescent complex that acts as an acceptor. The short linker (~9 amino acids) allows efficient FRET from GFP to the activated FAST-fluorogen complex, leading to a decrease in GFP fluorescence lifetime, detectable by **FLIM (Fluorescence Lifetime Imaging Microscopy)**. **After washing out the fluorogen (bottom):** The FAST-fluorogen complex dissociates, resulting in the loss of FRET from GFP to FAST. Consequently, the GFP fluorescence lifetime returns to its normal value.

**Supplementary Figure 8. Absorption and fluorescence emission spectra of GFP and fluorogens-bound-FAST complexes.** GFP absorption and emission spectra are shown together with the absorption emission spectra of FAST bound to HMBR, HBR-3,5-DM, and HBR-3,5-DOM. The progressive red shift in fluorogen emission from HMBR to HBR-3,5-DOM increases the spectral separation from GFP emission and influences the donor–acceptor spectral overlap relevant for FRET measurements. All spectra are normalized to their respective maxima for comparison.

**Supplementary tables and legends**

**Supplementary Table 1. Crystallographic Data Collection Statistics**

The table summarizes the X-ray diffraction data collection parameters for the FAST-HBR-3,5-DM complex (FAST_DM) and the apo FAST structure (FAST_apo).

**Supplementary Table 2. Refinement and Model Validation Statistics**

The table presents the refinement results and validation metrics for the FAST-HBR-3,5-DM complex and apo FAST structures.

**Supplementary Table 3. Fluorescence Lifetime Analysis of Soluble FAST in Live Neuro-2a Cells Across Varying Fluorogen Concentrations.** The median (IQR) and mean ± SD fluorescence lifetime values of soluble FAST constructs measured in live Neuro-2a cells in the presence of 1.25 to 10 µM HMBR, HBR-3,5-DM and HBR-3,5-DOM are presented.

**Supplementary Table 4. Statistical Comparison of Mean Fluorescence Lifetimes of Subcellularly Targeted FAST Constructs in Live Neuro-2a Cells.** The p-values of mean fluorescence lifetime tested for significance with respect to soluble FAST, comparing Lyn-FAST, Mito-FAST, and Ensconsin-FAST constructs in live Neuro-2a cells are presented, in the presence of 10 µM HBR-3,5-DM (A) and in the presence of 10 µM HBR-3,5-DOM (B).

**Supplementary Table 5. Plasmids and primer sequences used for FAST cloning and mutagenesis.** All primer sequences are shown in the 5′–3′ direction.

**Supplementary Figures**

**Supplementary Figure 1. Ligand Interaction Maps of FAST with HBR-3,5-DOM and HMBR**

**Supplementary Figure 2. FLIM-Phasor Validation of Monoexponential Fluorophore Lifetimes of Rhodamine B and Coumarin 6**

**Supplementary Figure 3. Comparative FLIM Analysis of GFP and FAST–HMBR in Neuro-2a Cells**

**Supplementary Figure 4. Ligand Concentration Has Minimal Impact on FAST–Fluorogen Excited-State Dynamics**

**Supplementary Figure 5. Fluorescence Decay Analysis of GFP-Based FRET Constructs**

**Supplementary Figure 6. Reversible Fluorogen Labelling Enables Autonomous FRET Control with FAST**

**Supplementary Figure 7. Schematic representation of HBR-3,5-DOM binding to GFP-I-FAST and resulting FRET.**

**Supplementary Figure 8. Absorption and fluorescence emission spectra of GFP and fluorogens-bound-FAST complexes**

**Supplementary tables**

**Supplementary Table 1. Crystallographic Data Collection Statistics for Apo FAST and FAST Bound to HBR-3,5-DM**

**Supplementary Table 2. Crystallographic Refinement and Model Validation Statistics for Apo FAST and the FAST–HBR-3,5-DM Complex**

**Supplementary Table 3. Fluorescence Lifetime Analysis of Soluble FAST in Live Neuro-2a Cells Across Varying Fluorogen Concentrations**

**Supplementary Table 4. Statistical Comparison of Mean Fluorescence Lifetimes of Subcellularly Targeted FAST Constructs in Live Neuro-2a Cells**

**Supplementary Table 5. Plasmids and primer sequences used for FAST cloning and mutagenesis**
